# Research Participation in Inflammatory Bowel Disease Studies: What Do Patients Want?

**DOI:** 10.1093/crocol/otaf016

**Published:** 2025-02-27

**Authors:** Charles N Bernstein, Gia Ly, Zoann Nugent, Seth R Shaffer, Harminder Singh, Lesley A Graff

**Affiliations:** University of Manitoba IBD Clinical and Research Centre, Max Rady College of Medicine, Rady Faculty of Health Sciences, University of Manitoba, Winnipeg, MB, Canada; Department of Internal Medicine, Max Rady College of Medicine, Rady Faculty of Health Sciences, University of Manitoba, Winnipeg, MB, Canada; University of Manitoba IBD Clinical and Research Centre, Max Rady College of Medicine, Rady Faculty of Health Sciences, University of Manitoba, Winnipeg, MB, Canada; Department of Internal Medicine, Max Rady College of Medicine, Rady Faculty of Health Sciences, University of Manitoba, Winnipeg, MB, Canada; University of Manitoba IBD Clinical and Research Centre, Max Rady College of Medicine, Rady Faculty of Health Sciences, University of Manitoba, Winnipeg, MB, Canada; Department of Internal Medicine, Max Rady College of Medicine, Rady Faculty of Health Sciences, University of Manitoba, Winnipeg, MB, Canada; University of Manitoba IBD Clinical and Research Centre, Max Rady College of Medicine, Rady Faculty of Health Sciences, University of Manitoba, Winnipeg, MB, Canada; Department of Internal Medicine, Max Rady College of Medicine, Rady Faculty of Health Sciences, University of Manitoba, Winnipeg, MB, Canada; University of Manitoba IBD Clinical and Research Centre, Max Rady College of Medicine, Rady Faculty of Health Sciences, University of Manitoba, Winnipeg, MB, Canada; Department of Internal Medicine, Max Rady College of Medicine, Rady Faculty of Health Sciences, University of Manitoba, Winnipeg, MB, Canada; University of Manitoba IBD Clinical and Research Centre, Max Rady College of Medicine, Rady Faculty of Health Sciences, University of Manitoba, Winnipeg, MB, Canada; Department of Clinical Health Psychology, Max Rady College of Medicine, Rady Faculty of Health Sciences, University of Manitoba, Winnipeg, MB, Canada

**Keywords:** research participation, clinical trial, COVID pandemic, survey

## Abstract

**Background:**

We aimed to determine patient perspectives on inflammatory bowel disease (IBD) research participation and potential changes related to the COVID pandemic experience.

**Methods:**

Participants of the population-based University of Manitoba IBD Research Registry were surveyed March 2022 to March 2023. The survey inquired about views on IBD research participation in the pre-, peri- and post-COVID era. Questions included aspects of participation from home or in-person, potential reimbursement, results reporting, and study design. We determined a rank order of reasons for research participation. We assessed willingness to participate in 5 research genres: clinical trials, biospecimen collection research, research involving colonoscopies, research accessing medical records, and research with access to records and samples.

**Results:**

Of 3018 invitees, 1105 (36.6%) completed the survey. Two-thirds reported that pre-pandemic they were unlikely to participate in placebo-controlled clinical trials, and nearly half would participate in a trial if guaranteed to receive active drug. The most important aspect impacting on clinical trial participation was understanding the potential side effects (81%). Post-COVID, 20%-30% reported that their interest in research participation decreased, 15%-20% reported that their interest had increased, with the majority (55%-60%) indicating no change in research participation interest. About 80% would participate in observational research. Payment for participation was not a significant motivator for most.

**Conclusions:**

We found a low rate of interest in participating in placebo-controlled IBD clinical trial research but nearly 50% would participate in clinical trial research receiving active drug and 80% would participate in observational research. Research participation interest, however, was further lessened by the COVID pandemic.

**What is known**

The participation of patients with inflammatory bowel disease (IBD) in research studies is not robust. There are limited data as to the views of patients regarding their interests in participating in research studies, especially post-COVID pandemic which may have changed potential participants’ views on in-person participation.

**What is new here**

We found a relatively low rate of interest in participating in placebo-controlled IBD clinical trial research but nearly 50% would participate in clinical trial research receiving active drug and up to 80% would participate in observational research, depending on research type. Research participation interest, however, was lessened by the COVID pandemic. Awareness of research was low and was the most significant variable impacting participation.

## Introduction

The prevalence of inflammatory bowel disease (IBD) is rising worldwide. IBD can be debilitating and hence ongoing clinical research is imperative to improve disease understanding, prevention, and outcomes. Research to explore new therapies and optimize utilization of current therapies is necessary since current therapies have their limitations in terms of efficacy and safety.^1^ There is little question that more research is required to better manage this disease; however, there are several barriers to conducting human research studies; the 2 main ones being funding and patient participation.^2^ In fact, in a special meeting of the International Organization for the Study of Inflammatory Bowel Disease, a survey completed by the members ranked patient recruitment as the greatest challenge in designing and completing clinical trials.^3^ While experts have outlined important tenets to streamline clinical trial conduct,^3^ there remains a need to understand what approaches could enhance patient participation. Whether it is cost, participant recruitment, or other factors, it is notable that most large industry-funded randomized controlled trials (RCTs) have moved outside of North America and western Europe, and are obtaining study enrollment more commonly in Eastern Europe, Asia, and South America. It is anticipated that affected individuals would be highly motivated to participate in research considering IBD can have such a negative impact on quality of life, a risk for surgery, disability, and be associated with high direct and indirect costs. Further, IBD is heritable, which puts children of those with IBD at risk, potentially also serving as a motivator for affected parents to participate in research. However, many western research centers struggle to enroll participants in not only RCTs but even observational research. In an IBD partners survey study of nearly 15 000 participants, RCT participation declined from 1.1% of the cohort to 0.7% between 2011 and 2018, even though the number of available IBD RCTs doubled during that time period.^4^

Prior studies regarding research participation for IBD have provided some direction, but have generally been small and based on convenience samples. Two hundred persons with IBD attending the Massachusetts General Hospital (61% with Crohn’s disease [CD]), of whom 61% had participated in some type of research previously, were surveyed as to their interests and attitudes in participating in clinical trial research.^5^ Of persons who had previously participated in a research study, 50% were not interested in participating in another one. Only 15% of respondents were interested in participating in a study that required randomization, frequent visits, and a colonoscopy, all standard aspects of any quality random RCT of the past 30 years. The only characteristics that clearly differentiated between those willing and not willing to participate were gender and disease status, with men and those in an active disease flare more willing to participate.

A survey study of 226 persons from 6 countries found only about one-quarter of respondents had participated in a prior research study.^6^ The major barriers to participating in RCTs were invasive monitoring or screening (35%), concern over receiving placebo (35%), or suboptimal treatment (33%). Most respondents (68%) reported that clinical trial participants are “guinea pigs,” underscoring the negative connotations around clinical trial research.

The COVID-19 pandemic had many important impacts on healthcare delivery and health perceptions of the population, both positive and detrimental.^7^ The public became much more aware of infectious disease transmission, and much more reluctant to attend clinics and hospitals for clinical care.^8^ Many research trials were paused as nonessential activities.^8^ With the pandemic’s impact on usual healthcare delivery, it stands to reason it may also have impacted patient desire to participate in research. In the context of having experienced the COVID-19 pandemic, it is possible there was a growth in respect for research with more keenness to participate in research armed with the knowledge that it is research that helps tamp down disease. Further, out of necessity, healthcare delivery became virtual during the pandemic and much has remained virtual post-pandemic, which has been seen as beneficial to improve access.^9,10^ Alternatively, especially among those with chronic disease and immune compromise such as individuals with IBD, the pandemic experience may have escalated fears of infection transmission, with clinics and hospitals considered unsafe environments, undermining interest in research opportunities. Undoubtedly, there are lessons to be learned from healthcare delivery and the conduct of clinical trials during the pandemic that will inform clinical research conducted post-pandemic^11^

In a large population-based sample of adults with IBD, we aimed to examine patient perspectives on IBD research participation both for RCTs and for observational research, including changes from the pre- to post-pandemic period, using a retrospective approach. We hypothesized that persons with IBD would have greater interest in participating in clinical research, both RCT and observational, post pandemic but that they would prefer more remote/virtual participation.

## Methods

The University of Manitoba IBD Research Registry is a population-based registry with 3018 adult registrants with up-to-date contact information, which included either e-mail addresses or home addresses. All were sent the survey on research engagement and invited to participate. Two subsequent contacts were made to prompt participation for nonresponders. Consent was obtained from all participants. The survey and study protocol were approved by the University of Manitoba Health Research Ethics Board.

The survey was created in the spring of 2022 as the COVID pandemic was nearing its end. Survey distribution occurred in March 2022 through March 2023 (for all questions, see Supplementary Table). It was apparent to our research team that it was more challenging to enroll participants in studies that required in-person visits to our hospital or clinic settings. Hence, we designed a survey that inquired about views on research study participation in the pre-pandemic era and during the COVID pandemic era to identify and potentially mitigate barriers. The survey included questions of views of participating without the concerns for the risks associated with a pandemic. The survey included questions about clinical treatment trial participation and observational study participation, and considerations for at-home or in-person participation, potential reimbursement, results reporting, and aspects of study design.

To determine the impact on clinical trial participation, the survey included temporally anchored questions such as: “Before the COVID-19 pandemic, how likely were you to participate in the following types of clinical trials for the treatment of IBD?,” “During the COVID-19 pandemic, how did your interest change in participating in the following types of clinical trials for the treatment of IBD?,” “Imagine that the risk of contracting COVID-19 has significantly decreased: compared to right now, how likely would you be to participate in the following types of clinical trials for the treatment of IBD?” Response options ranged from 1 = decreased significantly or significantly less likely to 5 = increased significantly or significantly more likely. For these questions the answer choices of somewhat and significantly increased or more likely were grouped, and somewhat and significantly reduced or less likely were grouped. For the question: “If you were to participate in a clinical trial for the treatment of IBD in the next 6 months, assuming COVID-19 is still posing a risk for serious disease, how important are the following on your interest in participating?” the answer choices were most important, least important, neither for each of the potential factors (eg, access to newest therapies). Parallel questions were asked in relation to participating in observational research studies. These questions were derived through patient input obtained from those presenting for routine clinic as well as for clinical research.

People who responded to at least 1 question with “somewhat likely” or “very likely” to participate where classified as the “willing” group and the other respondents were considered as the comparator “less/not willing” group. The data were analyzed by determining response distribution by percentage. For persons who expressed a level of willingness to participate in either treatment trial research or observational research, logistic regression was used to identify predictive demographic characteristics for participation, examining sex, age, income, whether working or not working (retired, on disability), education and awareness, presence of depression or anxiety, and opinions about trials. To determine the rank order of reasons for participating in research, the 5 most frequently endorsed responses were scored 5 to 1 in descending order for each respondent, and then all reasons’ scores were summed and a mean number derived. For individuals, their “reason to take part” was the reason ranked as most important, that is, all the participants were assigned to the group for logistic analyses, determined by their individual most important reason.

We assessed responses reflecting willingness to participate regarding 5 types of research participation (clinical drug trials, biospecimen sample collection research, research involving colonoscopies, research accessing medical records, and research accessing records and samples). We undertook a logistic regression analysis to determine if there were differing predictors of participation depending on the type of research.

In relation to these 5 types of research designs, participants were also surveyed to determine if their willingness to take part in the various research designs pandemic had changed during the pandemic, and whether an environment with an assured low COVID risk would affect participation compared to prior to the COVID pandemic. These responses were modeled in a similar regression analysis, controlling for willingness to participate pre-COVID. Group membership (willing to participate/less willing) was used as a dichotomous dependent variable in a logistic regression with demographic and opinion data in the models. Changes in attitude were dichotomized (more likely vs less likely) and analyzed with the same model with the added factor of the original attitude of willing or less willing.

## Results

One-third (36.6%) of the IBD registry, 1105 persons completed the survey of which nearly 60% were female, 83% were over age 40 (median age = 60, interquartile range 47-68), and over 75% had at least some college education. Close to half were working or students, and nearly 42% were retired. Half of the respondents had CD, with the remainder having ulcerative colitis (Table 1). The most common comorbidities were anxiety (21%), hypertension (20%), and depression (16%).

**Table 1.** Demographics

Gender	
Male	41.1%
Female	58.4%
Non binary	0.5%
Race	
White	90.7
Aboriginal/First Nation/Inuit	4.8 %
Other	4.5%
Age	
≤20	2%
21-30	2%
31-40	10%
41-50	13%
51-60	23%
61-70	29%
71+	18%
missing	2%
Education	
Less than high school degree	4.7%
High school degree or equivalent	18.1%
Some college but no college degree	16.7%
College/university degree	41.7%
Postgraduate degree	11.7%
Professional degree (eg, MD, JD, etc.)	6.3%
Prefer not to say	0.7%
Income	
$10,000-$29,999	6.7%
$30,000-$49,999	11.1%
$50,000-$69,999	12.2%
$70,000-$89,999	12.5%
$90,000-$109,999	11.2%
$110,000-$149,999	10.9%
$150,000+	18.7%
Prefer not to say	16.7%
Employment	
Full-time work	38.5%
Part-time work	9.3%
Full-time student	1.6%
Part-time student	0.3%
Unemployed and seeking work	1%
Unemployed and not seeking work	1.2%
Retired	41.8%
On disability insurance	4.4%
Stay at home parent	1.2%
n/a	0.7%
IBD type	
UC	46.3%
CD	54.9%
IBD—Other	5.2%
Comorbidities	
Diabetes	9.8%
Asthma/COPD	9.4%
Hypertension	20.8%
Cirrhosis	0.9%
Chronic kidney disease	2.4%
Ischemic heart disease/past heart attack/cardiac stent/arrhythmia	4.0%
Stroke	1.7%
Depression	16.2%
Anxiety	21.5%
None	39.1%
Other	20.5%
Research trial knowledge	
How aware are you of clinical treatment trials?	
Very aware	24.6%
Somewhat aware	49.9%
Not aware	25.5%
How many research clinical trials have you participated in?	
0	67.5%
1	13.4%
2	7.0%
3	3.4%
4 or more	8.7%

One-quarter of respondents were unaware of clinical treatment studies. Nearly 20% of respondents had participated in 2 or more clinical research trials (Table 1). Twenty percent was more than anticipated and hence, since it was asked early in the survey, it is unclear if respondents were answering in regards to participating in any kind of research study.

Almost half of participants were willing to participate in clinical research even if they never received results. More important than getting the results was ensuring good clinical care was met. The vast majority preferred to receive study results by regular mail or e-mail, rather than in person.

The reasons to participate in clinical research which were most highly endorsed were: “I want to contribute to medical science and enhance knowledge about my disease to help patients after me” (3.36), “I want to improve my quality of life” (3.13), “I want to receive the most up-to-date therapies without high expense” (2.00), “I want to access nurses/doctors who are experts in their field” (1.99), and “I want to try something new to address my disease” (1.26).

### Clinical Trial Participation

#### Pre-pandemic period

When asked about the likelihood before the COVID-19 pandemic to participate in different types of clinical trials for the treatment of IBD, 55% reported being somewhat or very likely to participate in a trial where samples were collected, only 34% were somewhat or very likely to participate in placebo-controlled trial, 48% were somewhat or very likely to participate in a trial where all participants received active drug, and 28% were somewhat or very likely to participate in a trial involving colonoscopy at baseline and at 4 months (Supplementary Table).

Close to half (46%) of participants indicated that prior to the pandemic they were unlikely to participate in a clinical trial that involved placebo. Of those only 19% said they were likely or very likely to participate in trials where the participant was guaranteed to receive active drug. Overall, only 35% would attend monthly clinic visits as part of the trial, but 53% would attend initial and final visits at a clinic with all other visits conducted remotely. Half of respondents were unlikely to participate in a trial with colonoscopy scheduled at baseline and at either 4 or 12 months later. The question did not allow for discerning whether the reluctance was secondary to the number of colonoscopies or frequency of colonoscopies.

In terms of the parameters that are most important to potential participants in considering whether to engage in IBD clinical trial research, 81% reported an understanding of potential side effects, 62.2% reported a clear understanding of what is expected of them, 58.6% reported the time commitment expected, 51.4% reported that a doctor or nurse was available for support at some or all check-ins and 48%-49% reported samples being shipped to their home or samples being collected at home. Reimbursement for time and/or travel was prioritized for only a small proportion.

#### During the pandemic

**Figure 1.**
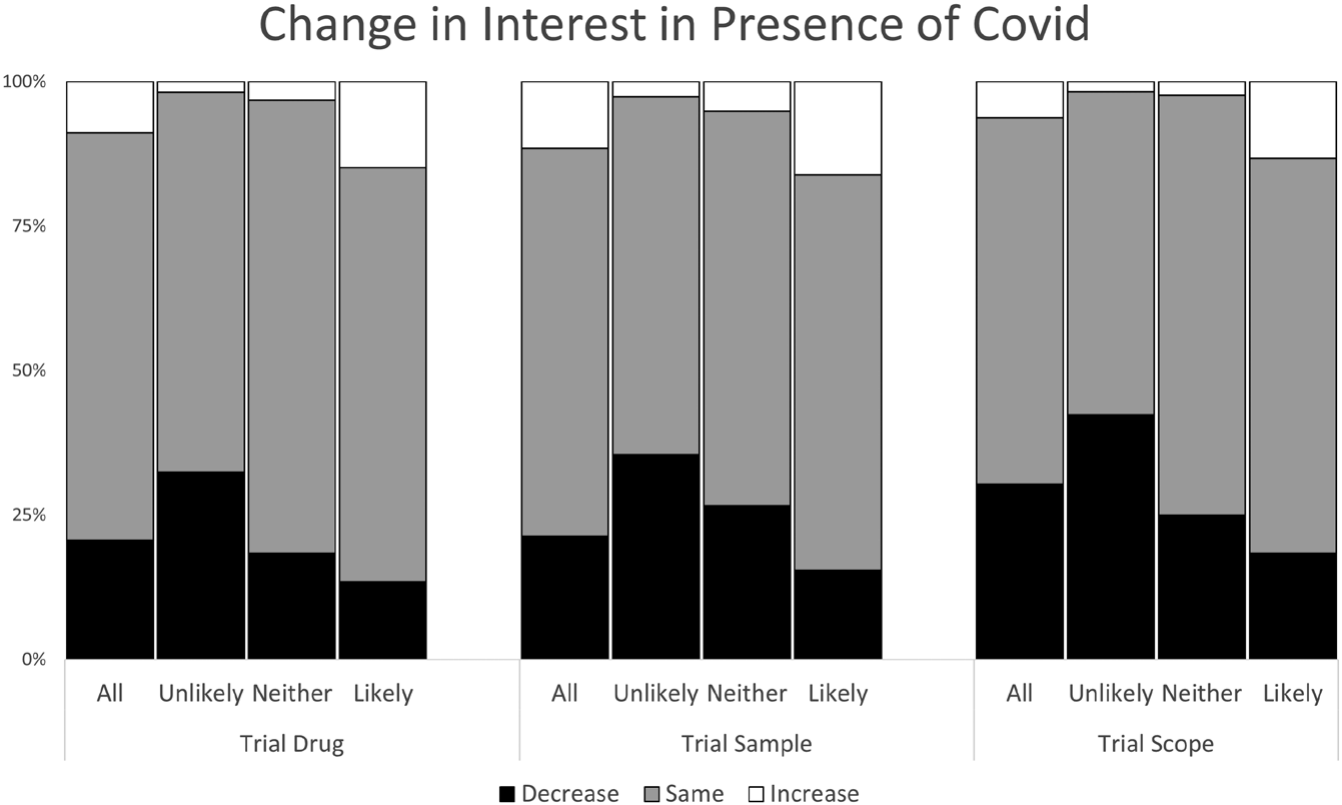
The impact of COVID pandemic on interest in participating in a clinical drug trial (Trial Drug), in a study where biological samples are collected (Trial Sample), or in a study that included colonoscopy (Trial Scope) The categories refer to all participants, those who were unlikely to participate pre-pandemic, those who were likely to participate pre-pandemic and those who were neither unlikely or likely to participate.

Regardless of the clinical trial parameters approximately 20%-30% reported that their interest in participating in IBD research decreased either somewhat or significantly during the pandemic. Approximately 6%-12% reported that their interest increased somewhat or significantly (Figure 1), and overall most reported there was no change in their interest.

#### Post-pandemic period

**Figure 2.**
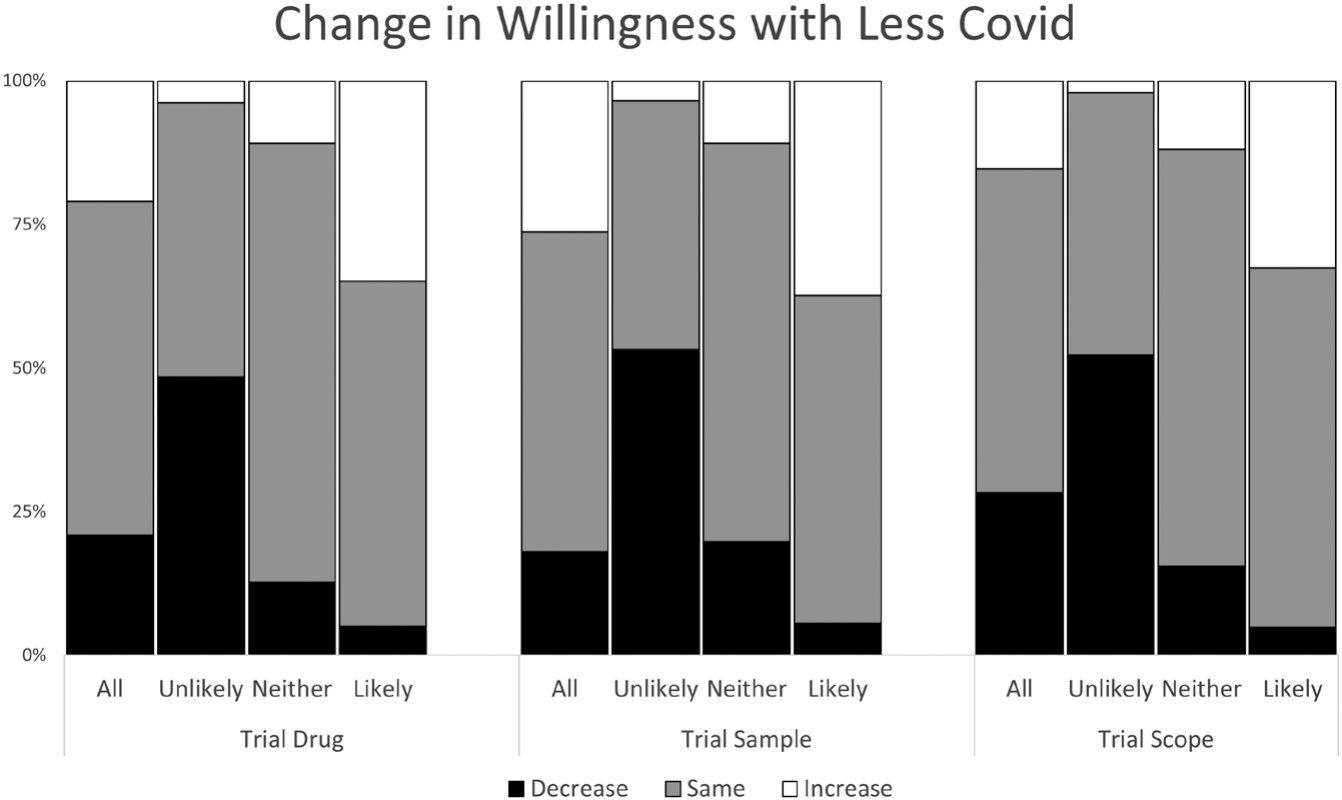
The impact of the resolution of COVID pandemic on willingness to participate in a clinical drug trial (Trial Drug), in a study where biological samples are collected (Trial Sample), or in a study that included colonoscopy (Trial Scope) The categories refer to all participants, those who were unlikely to participate pre-pandemic, those who were likely to participate pre-pandemic, and those who were neither unlikely or likely to participate.

Regardless of the trial parameters, approximately 15%-25% reported that their interest in participation would either somewhat or significantly increase. However, despite the reduced COVID risk in the post-pandemic period, 20%-30% reported adecreased interest in participation (Figure 2).

### Predictors for Participation in Clinical Trials Prior to COVID Pandemic

For participation in clinical trials, not surprisingly, having no awareness of trials was a strong predictor of reduced interest in participating (OR 0.51, 95% CI, 0.33, 0.71), and having previously participated in a clinical trial significantly increased the likelihood of participation (OR 1.65, 95% CI, 1.21, 2.26). Being somewhat aware of clinical trials reduced the likelihood of participating but was only statistically significant in studies requiring colonoscopy. Participants giving any reason other than increasing medical knowledge (reference group for logistic regression) were either not significantly different from this reference or less likely to participate.

Similarly, those endorsing “doctor recommendation” were less likely to participate relative to those where the primary reason was increasing medical knowledge. “Access to experts” as a primary reason was not associated with increased participation. Being concerned about receiving research results did not enhance participation in clinical trials (Table 2a). There were no demographic variables including age, sex, income, retirement vs working status, or presence of comorbidities or depression/anxiety.

**Table 2.** (a) Logistic analyses modeling responses of “likely” or “very likely” to participate in clinical trials; (b) logistic analyses modeling responses of “likely” or “very likely” to participate in observational research.

(a)						
Type	Clinical drug trial		Clinical drug trial with biospecimen collection research		Clinical drug trial research including colonoscopy	
% likely or very likely	51		64		38	

Predictors	OR (95% CI)	P	OR (95% CI)	P	OR (95% CI)	P
Age	1.01 (0.99-1.02)	.30	1.01 (0.99-1.03)	.31	1.00 (0.98-1.02)	.92
IBD						
Other	0.95 (0.59-1.55)	.84	0.83 (0.49-1.4)	.48	0.85 (0.52-1.40)	.52
UC	1.00 (0.75-1.34)	.99	0.78 (0.57-1.06)	.11	1.21 (0.90-1.63)	.21
CD	ref		ref		ref	
Education	1.00 (0.89-1.14)	.95	1.04 (0.91-1.18)	.61	1.06 (0.94-1.21)	.34
Income	0.98 (0.91-1.07)	.71	1.09 (0.99-1.19)	.068	0.99 (0.91-1.08)	.88
Awareness						
Very	ref		ref		ref	
Somewhat	0.72 (0.51-1.03)	.072	0.77 (0.52-1.13)	.19	**0.69 (0.49-0.98)**	**.037**
None	**0.51 (0.33-0.77)**	**.0015**	**0.46 (0.3-0.72)**	**.0007**	**0.54 (0.35-0.82)**	**.0044**
Previous trials (Y vs N)	**1.65 (1.21-2.26)**	**.0018**	**1.58 (1.12-2.22)**	**.0097**	1.32 (0.97-1.8)	.081
Reason						
Access to experts	0.88 (0.43-1.81)	.72	0.62 (0.3-1.3)	.2066	0.47 (0.2-1.09)	.077
Doctor recommendation	0.51 (0.24-1.06)	.069	0.34 (0.16-0.7)	.0037	0.59 (0.27-1.28)	.18
Improve my life	1.21 (0.86-1.70)	.28	1 (0.7-1.45)	.99	1.01 (0.72-1.43)	.96
Newest therapies	1.14 (0.6-2.17)	.68	0.57 (0.29-1.1)	.0909	1.11 (0.59-2.1)	.75
New-to-me therapies	1.11 (0.53-2.33)	.79	1.11 (0.49-2.51)	.81	1.43 (0.69-2.96)	.33
More tests	1.00 (0.42-2.41)	1.00	1.72 (0.6-4.96)	.31	1.53 (0.63-3.71)	.35
Other	**0.39 (0.20-0.76)**	**.0056**	**0.51 (0.26-0.97)**	**.042**	0.65 (0.33-1.27)	.21
Increase knowledge	ref		ref		ref	
Male	1.22 (0.91-1.62)	.18	0.81 (0.60-1.10)	.18	1.28 (0.96-1.71)	.10
Receiving results						
Eventually	0.86 (0.62-1.19)	.37	**0.68 (0.48-0.96)**	**.029**	**0.69 (0.5-0.96)**	**.027**
within 6 months	0.67 (0.4-1.15)	.15	**0.47 (0.27-0.82)**	**.0077**	0.74 (0.43-1.28)	.27
within 1 year	0.8 (0.53-1.22)	.31	**0.61 (0.39-0.94)**	**.026**	**0.6 (0.39-0.93)**	**.022**
don’t care	ref		ref		ref	
Comorbidities (Y vs N)	0.99 (0.73-1.34)	.95	0.86 (0.62-1.19)	.37	0.99 (0.73-1.35)	.97
Depression/anxiety (Y vs N)	0.92 (0.67-1.27)	.61	1.28 (0.9-1.81)	.17	1.07 (0.77-1.48)	.69
Employment						
Other vs full-time	0.81 (0.54-1.22)	.32	1.13 (0.73-1.75)	.58	1.46 (0.97-2.19)	.072
Retired vs full-time	0.9 (0.58-1.38)	.63	1.07 (0.68-1.69)	.77	1.26 (0.81-1.94)	.30

(b)						
Type	Research using health records		Research using records & specimens			
% likely or very likely	85		71			

Predictors	OR (95% CI)	P	OR (95% CI)	P		
Age	1.00 (0.97-1.02)	.68	1.00 (0.98-1.02)	.88		
IBD						
Other	1.11 (0.50-2.46)	.80	0.89 (0.51-1.54)			
UC	0.81 (0.52-1.27)	.37	0.84 (0.61-1.17)	.30		
CD	ref		ref			
Education	1.05 (0.87-1.27)	.59	1.01 (0.88-1.16)	.87		
Income	1.02 (0.90-1.16)	.74	1.02 (0.93-1.12)	.67		
Awareness						
Very	Ref		ref			
Somewhat	**0.47 (0.25-0.87)**	**.017**	0.90 (0.60-1.35)	.61		
None	**0.31 (0.16-0.63)**	**.0011**	**0.62 (0.39-0.98)**	**.041**		
Previous trials (Y vs N)	**0.60 (0.37-0.95)**	**.030**	1.2 (0.84-1.71)	.33		
Reason						
Access to experts	0.53 (0.22-1.29)	.16	0.98 (0.45-2.12)	.96		
Doctor recommendation	0.98 (0.35-2.76)	.96	0.49 (0.24-1)	.051		
Improve my life	0.98 (0.57-1.67)	.94	1.13 (0.76-1.66)	.55		
Newest therapies	0.76 (0.30-1.89)	.55	1.01 (0.49-2.07)	.98		
New-to-me therapies	0.82 (0.26-2.51)	.72	1.10 (0.47-2.56)	.83		
More tests	1.16 (0.25-5.34)	.85	1.01 (0.38-2.74)	.98		
Other	0.54 (0.23-1.30)	.17	0.80 (0.40-1.60)	.53		
Increase knowledge	ref		ref			
Male	0.75 (0.49-1.16)	.20	0.84 (0.61-1.16)	.29		
Receiving results						
eventually	0.70 (0.41-1.18)	.18	0.73 (0.50-1.06)	.094		
within 6 months	**0.28 (0.15-0.54)**	**.0002**	**0.39 (0.23-0.68)**	**.0009**		
within 1 year	0.60 (0.32-1.13)	.11	**0.57 (0.36-0.90)**	**.015**		
don’t care	ref		ref			
Comorbidities (Y vs N)	0.93 (0.59-1.47)	.75	0.96 (0.68-1.34)	.79		
Depression/anxiety (Y vs N)	0.70 (0.44-1.12)	.14	0.83 (0.58-1.18)	.30		
Employment						
Other vs full-time	1.62 (0.81-3.26)	.18	1.44 (0.90-2.30)	.13		
Retired vs full-time	1.08 (0.56-2.1)	.81	1.17 (0.72-1.88)	.53		

When asked about reimbursement for 1-year clinical trial participation with on-site visits, as an open-ended question the mean amount suggested was $2006, and the median amount was $24. For a 1-year clinical trial that was all undertaken remotely the mean reimbursement amount suggested was $236 but the majority reported no reimbursement would be necessary.

### Participation in Observational Clinical Research

Participants were asked to exclude their involvement in this survey study when responding—75% indicated they have participated in observational clinical research in the past, which encompasses measurement through surveys, clinical interviews, and sample collection (eg, blood, saliva, stool or tissue, colonoscopy).

#### Pre-pandemic period

Eighty percent indicated they would likely allow their health records to be reviewed, or to complete surveys virtually/remotely. Fifty percent to 60% reported they would be likely to provide biological samples on site or at home, while 20%-25% felt they would not allow for biological sampling regardless of site.

#### During the pandemic

During the pandemic, the interest in participating in observational research and in having biological sampling did not change (Figure 1).

#### Post-pandemic period

If the risk of COVID was to decrease, approximately 20% reported that their interest in participating in observational research including with biological sampling would increase. For the majority, their interest did not change even when considering a reduction in COVID risk (Figure 2).

### Predictors for Participation in Observational Clinical Research Prior to COVID Pandemic

Having no awareness of trials was a strong predictor of reduced likelihood to participate in biospecimen collection research (OR = 0.62, 95% CI, 0.31-0.98), and in research requiring colonoscopy (OR = 0.53, 95% CI, 0.35, 0.81). Having previously participated in a clinical trial did not impact on the likelihood of participation in biospecimen collection research (OR = 1.2, 95% CI 0.84, 1.71, Table 2a). Participation in research using health records was less likely if unaware of previous research or even somewhat aware (vs very aware) (Table 2b). Health record research with biospecimen collection was less likely, if unaware of the research opportunities, which of course is a very clear barrier (Table 2b). Previous participation reduced the likelihood of taking part in records-based research and had no significant impact if biospecimen collection was involved. There were no demographic variables including age, sex, income, retirement vs working status, or presence of comorbidities or depression/anxiety that predicted observational research participation. There was a weak association between interest in payment and willingness to take part in research. (Table 3).

**Table 3.** Attitudes regarding participation and payment.

Trial type	Attitude to participation	Payment for clinical trial (%)			P
		No	Gas & parking	Yes	
Drug	Unlikely	38	5	58	.041
	Neither	35	7	58	
	Likely	29	8	63	
Biospeciman collection	Unlikely	36	3	61	.19
	Neither	42	7	51	
	Likely	30	8	62	
Colonoscopy inclusion	Unlikely	34	5	61	.59
	Neither	36	9	55	
	Likely	30	8	62	

		Payment for remote trial (%)			
		No		Yes	
Health records	Unlikely	53		47	.62
	Neither	49		51	
	Likely	49		51	
Health records & specimens	Unlikely	55		45	.21
	Neither	49		51	
	Likely	48		52	

## Discussion

In this large, population-based study of adults with IBD there was a surprising level of reluctance or disinterest in participating in placebo-controlled clinical research; however, nearly 1 in 2 persons would participate in clinical trial research with guarantee to get active drug and up to 80% would participate in observational research depending on the type. Respondents reported that prior to the COVID-19 pandemic, 55% would have participated “in a trial where biosamples were required”; however, less than 50% would have participated in other types of research, including only 34% in a placebo-controlled trial and 48% in a trial where all participants received active drug. The study found that there was a greater willingness to participate in observational research relative to clinical trials. In general, the end of the COVID pandemic was not seen to enhance research participation by persons with IBD. Financial reimbursement was not a strong incentive to attract participation. Remote (ie virtual) participation was of interest to a large number of participants. Interestingly, receiving results of the study was not critical for almost half (45%) of participants, although it was especially relevant for research involving biospecimen collection and colonoscopies.

Among the 5 top reasons to participate in research the primary motivator was an altruistic one, namely to contribute to medical science and enhance disease knowledge and help future patients. Improving quality of life was the next most important reason. Accessing most up-to-date therapies and more knowledgeable healthcare providers was important but lower on the list of reasons to participate. In terms of factors that are important in the conduct of clinical trials, 4 out of 5 (81%) reported that an understanding of potential side effects was important. Hence, while clinical trials are undertaken in patients who are typically unwell and drugs are studied to achieve remission, or alternatively to maintain remission, understanding the potential side effects is paramount to patients. Since there is always some uncertainty regarding safety profiles in drugs or procedures that are undergoing exploratory studies, this may be a significant dissuading factor for participation, and may require additional attention in patient recruitment.

Forty to fifty percent of respondents were simply not interested in participating in clinical trial research. While there has been much discussion about how to enhance clinical trial participation and also the degree to which clinical trials reflect the general IBD population,^12^ there has yet to be a large study exploring patient impressions and influences about participating in research. While the possibility that only 31% of a specialty IBD clinic population is eligible for a clinical trial raising generalizability questions,^12^ the fact that up to half of a population-based sample would simply not want to participate suggests that persons enrolled in clinical trials may be a skewed and even less representative sample than previously considered. Might it be that those who would participate in clinical research have a different psychological makeup that, for instance, might enhance placebo response rates? When placebo response rates of 20% are seen in rigorous modern clinical trials does that reflect a selected interested population, keen on contributing broadly to disease knowledge? Our results might even overestimate interest in research participation since the respondents were recruited from a research registry, which in itself suggests some receptivity, and 1 in 3 in the Registry self-selected to participate in this survey study, raising concerns that the majority who did not participate in a straightforward survey despite incentivization with a gift card would be even less likely to participate in clinical treatment trial or observational research.

It may be that different factors would drive participation in pediatric research, although undoubtedly risk aversion would be very high on the list. In a Manitoba study of 118 caregivers of children with IBD (median age 14), 49% responded that money would encourage research participation.^13^ Hence, financial incentives may differ by jurisdiction or participant characteristics. In a survey of 126 potential participants in hypertension research study, the greatest impediment to participation was a higher risk of adverse effects followed by higher risk of being or being assigned to placebo, followed by lower payments.^14^ These first 2 aspects regarding side effects and reluctance to take part in studies with a placebo control arm were relevant for our study as well. Interestingly, there was a trend toward a positive interaction between income and the influence of payment on willingness to participate, suggesting that wealthier patients were more likely to be influenced to participate by financial incentives. In the Massachusetts General Hospital study, 38% of 200 respondents reported that financial remuneration would influence participation in an RCT.^5^ In a survey study of 949 patients with IBD, monetary compensation had the strongest effect on enhancing participation in RCTs^15^

Factors which have been found to drive research participation have included personal and altruistic considerations, similar to the outcomes of the current study where increasing medical knowledge was the top reason for participating. In the Massachusetts General Hospital study, the survey presented 8 choices as to reasons to participate in a randomized trial were: personal health benefit, altruism, contribution to scientific knowledge, more access to care, trust in your physician, money, my next option is surgery, failure of all other medical therapies. Personal health benefits were the highest reported primary motivation for participating in a research study (30.5%). In a Danish study of 41 patients with cancer and 79 patients with IBD, the majority of both disease groups were willing participants in research studies because of wishes to get access to a new drug (78% and 86%, respectively) and to be more closely monitored (71% and 89%, respectively).^16^ A majority of respondents rated helping future patients as either very important or important (71% and 84%, respectively).

Our study was conducted in the aftermath of the COVID pandemic. The investigators were uncertain if the profound lack of knowledge about COVID at the start of the pandemic and the fact that research contributed dramatically to end the pandemic would entice persons with IBD to increase research study participation to advance medical science, or alternatively, if persons with IBD were going to be more disinterested in medical research, perhaps related to either fear or distrust of healthcare and medical science. When participants were asked to consider the period just prior to the COVID pandemic, just about one-third of respondents would participate in a study with a placebo arm, yet only an additional 10%-14% would participate in clinical trials if guaranteed to get an active drug. There was greater interest in participating in trials with remote visits rather than on-site visits and half of respondents were unlikely to participate in a trial with colonoscopy scheduled at 2 times. Thirty percent of respondents reported a decrease in likelihood of clinical trial participation during COVID and 20%-30% reported a decrease in research participation interest in the aftermath of the pandemic.

We found that survey participants who indicated they would not take part in research pre-pandemic were even “less interested” and “not likely” to participate in various types of research when considering the post-pandemic period. Hence, if persons with IBD were not likely going to participate in research of various types there was an even further negative impact of the COVID pandemic and even upon its conclusion there was no evident increased interested generated to participate in research. The general public heard much about research, the scientific process, and the need for strong research data to guide decisions that may impact day-to-day lives, especially during a viral pandemic. Nonetheless, the COVID pandemic seemed to lessen interest in research participation among individuals with IBD.

Designing studies without placebo arms, with the potential for very little harm, with participation virtually and/or from home, and including sample accrual at home might increase enthusiasm for clinical trial participation. While some participants need to trust that they will receive the results of the study, many did not feel it was important to receive the results. With the number 1 motivation for participation being contribution to medical knowledge for the next generation of patients, it may be important both at the recruitment step and in delivering results for researchers to inform participants how the study overall will/has advanced medical knowledge.

With regards to participation in observational clinical research, 75% of our cohort had participated in clinical research involving a range of methods such as completing surveys, and providing samples like blood, saliva, stool, or tissue at time of coloscopy. Health record review and remote survey completion are attractive to persons with IBD, perhaps because of lighter participant burden. Persons with IBD were more likely to provide biological samples if obtained in their homes (either for their convenience or possibly because of a distrust or fear of attending clinic sites). Financial incentives, especially for travel, did not seem to be as important or relevant for most participants. The COVID pandemic or its conclusion did not seem to overtly impact on participation in observational research, although it might have had an impact on the greater interest of design aspects such as at-home sample collection and study visits.

Our study has provided a large and rich data set of perceptions of persons with confirmed IBD regarding clinical research participation. While our cohort had over representation of older age persons, there is benefit to these data as the IBD population in North America is aging and the elderly with IBD not only are increasingly prevalent in IBD clinics but may have more available time to participate in research studies. There is a paucity of research on older adults and their interest in research participation. Persons over 60 make up less than 10% of clinical trial participation in IBD and hence understanding what may encourage their participation or what aspects discourage their participation is important.

One limitation of our study is that participants were enrolled through an IBD research registry and so by definition they were willing to, at a minimum, be contacted by a research team (enrollment in the University of Manitoba IBD Research Registry is voluntary and not automatic for clinic attendees). Further, those who completed the survey, by definition were undertaking research, even though some reported disinterest in participating in observational research studies. Hence, our findings are likely an overestimate of interest in research participation relative to a general community sample of persons with IBD. An additional important limitation is that the demographics of our respondents, who are on average older with a higher proportion of women, were more likely to be retired, and more likely to be White than a general community sample. However, when examining age and sex factors in this study through multivariable analysis, neither they nor any other demographic variables were found to impact likelihood to participate in research. Further, as our cohort demographic was mostly White, the interest of research participation by visible minorities is not well defined in our study. This is an important group to study since IBD is increasing among minority groups in North America including immigrant populations, and recruitment from different racial and ethnic backgrounds is a focus for industry-sponsored clinical trials, in particular. Nonetheless, we found no demographic variable to be predictive of participation in either clinical trial or observational research. In fact, even the presence of depression or anxiety or even work status versus retirement status did not impact on research participation interest. Another important limitation is that in the immediate aftermath of the COVID pandemic we asked persons to consider their attitudes toward research prior to and during and even for the future when COVID would no longer pose a threat. It is certainly possible that a study on research participation undertaken prior to a major world health crisis and repeated after the health crisis would have a sharper read on perception changes without having to rely on retrospective recall, and may have provided different responses. Prospective longitudinal studies typically provide richer data. Nonetheless, this large cross-sectional study should encourage clinical researchers to determine the ways to optimize participation of the broadest representation of patients, to, in turn, optimize the extent to which any study can be generalizable.

Based on our study we believe that clinical trial research study design is going to need to change, particularly with elimination of placebo arms where standard of care includes effective drugs, and with reduced on-site participation. While regulatory authorities are keen that main trial endpoints reflect participant-reported outcomes they^17–20^ should also consider patient views on trial participation. The 1 significant predictor of research study participation was knowledge of research studies and hence, IBD research centers and patient support groups such as Crohn and Colitis Foundation may need to do a better job educating their IBD communities as to the importance of research studies and what research studies are ongoing.

## Supplementary Material

otaf016_suppl_Supplementary_Tables

## Data Availability

The data underlying this article are available in the article and in its online Supplementary Material. Hence, they are not otherwise not readily available.
