# Supplementary material for: Research Participation in Inflammatory Bowel Disease Studies: What Do Patients Want?
Source: Crohns Colitis 360. 2025 Feb 27;7(2):otaf016. doi: 10.1093/crocol/otaf016 (PMC12079376; doi:10.1093/crocol/otaf016)
Supplement: otaf016_suppl_Supplementary_Tables [file otaf016_suppl_supplementary_tables.docx]

**Patients’ experience with and perception of clinical treatment trials**

| How aware are you of clinical treatment trials? |  |
| --- | --- |
| Very aware | 24.6% |
| Somewhat aware | 49.9% |
| Not aware | 25.5% |
|  |  |
| How many research clinical trials have you participated in? |  |
| 0 | 67.5% |
| 1 | 13.4% |
| 2 | 7.0% |
| 3 | 3.4% |
| 4 | 8.7% |
|  |  |
| Can you estimate the usual number of doctor or health care provider visits you have in any year (please list number of visits) | Mean = 6.6 days |
| What is the maximum amount of in-person health care contact you be willing to have to participate in a clinical trial? Please select one of the following: |  |
| None | 12.8% |
| Same as usual | 34% |
| 1-5 more | 27.9% |
| 6-10 | 4.4% |
| As many as required | 20.9% |
|  |  |
|  |  |
|  |  |

**Before COVID-19, how likely were you to participate in the following types of clinical trials for the treatment of IBD?**

|  | Somewhat and Extremely likely | Neutral | Somewhat and Extremely Unlikely |
| --- | --- | --- | --- |
| Trial with a placebo arm (placebo is an inactive treatment), meaning, for example, there is a 1 in 3 or 1 in 5 chance to get an inert drug for some period of time, though all participants will eventually receive the study drug | 34.1% |  |  |
| Trial comparing two different drugs (each one possibly effective in treating inflammatory bowel disease) and you are randomized to receive one of the drugs | 40.5% |  |  |
| Single drug trial where all participants receive the active drug | 48.4% |  |  |
| Trial where samples (e.g., blood, stool, urine, etc.) are required | 54.7% |  |  |
| Trial requiring monthly on-site visits to the clinic or hospital site | 34.8% |  |  |
| Trial requiring a visit to the clinic or hospital site at the beginning and end of the trial, with all other visits conducted in- person at your home | 48.9% |  |  |
| Trial requiring a visit to the clinic or hospital site at the beginning and end of the trial, with all other visits conducted completely remotely (i.e., virtual visits) | 52.6% |  |  |
| A trial with a colonoscopy conducted at the beginning and end, lasting 4 months | 28.8% |  |  |
| A trial with a colonoscopy conducted at the beginning and end, lasting 1 year | 37.6% |  |  |
|  |  |  |  |

**During COVID-19, how did your interest change in participating in the following clinical trials for the treatment of IBD?**

|  | Increased somewhat and significantly |  |  |
| --- | --- | --- | --- |
| Trial with a placebo arm (placebo is an inactive treatment), meaning, for example, there is a 1 in 3 or 1 in 5 chance to get an inert drug for some period of time, though all participants will eventually receive the study drug | 5.2% |  |  |
| Trial comparing two different drugs (each one possibly effective in treating inflammatory bowel disease) and you are randomized to receive one of the drugs | 5.7% |  |  |
| Single drug trial where all participants receive the active drug | 8.1% |  |  |
| Trial where samples (e.g., blood, stool, urine, etc.) are required | 7.6% |  |  |
| Trial requiring monthly on-site visits to the clinic or hospital site | 5.3% |  |  |
| Trial requiring a visit to the clinic or hospital site at the beginning and end of the trial, with all other visits conducted in-person at your home | 6.4% |  |  |
| Trial requiring a visit to the clinic or hospital site at the beginning and end of the trial, with all other visits conducted completely remotely (i.e., virtual visits) | 9.3% |  |  |
| A trial with a colonoscopy conducted at the beginning and end, lasting 4 months | 5.2% |  |  |
| A trial with a colonoscopy conducted at the beginning and end, lasting 1 year | 6.2% |  |  |

**Imagine that the risk of contracting COVID-19 has significantly decreased: compared to right now, how likely would you be to participate in the following types of clinical trials for the treatment of IBD?**

|  | Increased somewhat and significantly |  |  |
| --- | --- | --- | --- |
| Trial with a placebo arm (placebo is an inactive treatment), meaning, for example, there is a 1 in 3 or 1 in 5 chance to get an inert drug for some period of time, though all participants will eventually receive the study drug | 14.6% |  |  |
| Trial comparing two different drugs (each one possibly effective in treating inflammatory bowel disease) and you are randomized to receive one of the drugs | 16.9% |  |  |
| Single drug trial where all participants receive the active drug | 20.3% |  |  |
| Trial where samples (e.g., blood, stool, urine, etc.) are required | 19.2% |  |  |
| Trial requiring monthly on-site visits to the clinic or hospital site | 17.2% |  |  |
| Trial requiring a visit to the clinic or hospital site at the beginning and end of the trial, with all other visits conducted in-person at your home | 19.2% |  |  |
| Trial requiring a visit to the clinic or hospital site at the beginning and end of the trial, with all other visits conducted completely remotely (i.e., virtual visits) | 20.8% |  |  |
| A trial with a colonoscopy conducted at the beginning and end, lasting 4 months | 11.6% |  |  |
| A trial with a colonoscopy conducted at the beginning and end, lasting 1 year | 15% |  |  |

**If you were to participate in a clinical trial for the treatment of IBD in the next 6 months, assuming COVID-19 is still posing a risk for serious disease, how important are the following on your interest in participating?**

|  | Most Important | Least Important | Neither |
| --- | --- | --- | --- |
| Clear understanding of side effects | 80.6% | 2% | 17.4% |
| Clear understanding of what is expected of me | 62.2% | 6.9% | 30.9% |
| Free medical care | 42.1% | 22.1% | 35.8% |
| 24-hour support, either virtual or by phone | 41% | 20.8% | 38.3% |
| Time commitment (i.e., participation will not take time away from obligations) | 58.6% | 10.1% | 31.3% |
| Reimbursement for time / travel | 28.7% | 28.5% | 42.8% |
| Ability to participate digitally / virtually (i.e., telemedicine) | 39.6% | 20% | 40.4% |
| There is direct support from a doctor or nurse for some or all of the check-ins | 51.4% | 13.2% | 35.4% |
| Medications shipped directly to my home | 48.2% | 12.8% | 38.9% |
| Samples collected at home and mailed to site | 49.2% | 12.7% | 38.1% |
| Access to trial location | 40.5% | 21.7% | 37.7% |
| Risk of COVID 19 exposure (i.e., whether location treats COVID 19 patients) | 44% | 21.2% | 34.7% |
|  |  |  |  |
|  |  |  |  |
|  |  |  |  |
|  |  |  |  |
|  |  |  |  |

**What do you view as the most important benefits of participating in clinical research (whether it is a clinical drug trial or other type of research such as survey or providing blood or other specimens)? Please select your top 5 and RANK in order of most important (top) to least important (bottom)**

|  | Ranked as most important |  |  |
| --- | --- | --- | --- |
| I want to contribute to medical science and enhance knowledge about my disease to help patients after me | 53% |  |  |
| I want to improve my quality of life | 34.8% |  |  |
| I want to receive the most up-to-date therapies without high expense | 7.4% |  |  |
| I want access to nurses / doctors who are experts in the field | 5.4% |  |  |
| Try something new to address my disease | 7.3% |  |  |
| I am following my doctors' recommendation | 15.3% |  |  |
| Getting more testing | 15.5% |  |  |
| Other | 50% |  |  |

"What level of financial reimbursement do you think is sufficient to participate in a 1 year clinical trial requiring on-site visits? Please list an amount that seems appropriate to you.

Mean = $2,006.42

Median = $24.00

Mode = $0.00

"What level of financial reimbursement do you think is sufficient to participate in a 1 year trial that is completely remote (i.e., virtual with no in-person visits) Please list an amount that seems appropriate to you.

Mean = $236.63

Median = $0.00 **more than half of values are 0*

Mode = $0.00

**The following questions will focus on your experience with and perception of clinical research. [Note: Clinical research does not include receiving a treatment in a trial. Types of clinical research include filling out surveys, providing samples like blood, saliva, stool or tissue at time of coloscopy, etc.]**

| Have you ever participated in clinical research (i.e., filling out surveys, providing samples like blood, saliva, stool or tissue at time of coloscopy, etc.). Do not consider your participation in this survey when answering this question. |  |
| --- | --- |
| Have participated | 74.6% |
| Have not participated | 25.4% |
|  |  |
| Outside of this survey, when did you last participate in clinical research? |  |
| Currently participating | 13.7% |
| Within last 6 months | 6.9% |
| 6-12 months | 11.1% |
| 1-2 years | 23.7% |
| 2-4 years | 11.4% |
| More than 4 years | 18.9% |
| I've never participated | 14.2% |

|  | Extremely or somewhat likely | Neither likely nor unlikely | Extremely or somewhat unlikely |
| --- | --- | --- | --- |
| **Before COVID-19, how likely were you to participate in the following types of clinical research for the treatment of IBD?** |  |  |  |
| Having your health care records accessed by a research team (i.e., no in-person / on-site contact required) | 80.5% |  |  |
| Filling out a survey remotely (i.e., no in-person / on-site contact required) | 81.9% |  |  |
| Providing biological samples in- person/on-site | 58.2% |  |  |
| Providing biological samples at home with in-person support from a healthcare provider (for example, an IBD nurse) | 54.5% |  |  |
| Providing biological samples remotely (i.e., mail-in, with virtual support) | 60.9% |  |  |
| **Imagine that the risk of contracting COVID-19 has significantly decreased: compared to right now, how likely would you be to participate in the following types of clinical research for the treatment of IBD** |  |  |  |
| Having your health care records accessed by a research team (i.e., no in-person / on-site contact required) | 22.5% |  |  |
| Filling out a survey remotely (i.e., no in-person / on-site contact required) | 21.9% |  |  |
| Providing biological samples in- person/on-site | 22.4% |  |  |
| Providing biological samples at home with in-person support from a healthcare provider (for example, an IBD nurse) | 17.8% |  |  |
| Providing biological samples remotely (i.e., mail-in, with virtual support) | 18.2% |  |  |
|  |  |  |  |

**During COVID-19, how did your interest changed in participating in the following types of clinical research for the treatment of IBD?**

|  | Increased somewhat and significantly |  |  |
| --- | --- | --- | --- |
| Having your health care records accessed by a research team (i.e., no in-person / on-site contact required) | 8.3% |  |  |
| Filling out a survey remotely (i.e., no in-person / on-site contact required) | 9.5% |  |  |
| Providing biological samples in- person/on-site | 6.4% |  |  |
| Providing biological samples at home with in-person support from a healthcare provider (for example, an IBD nurse) | 7.1% |  |  |
| Providing biological samples remotely (i.e., mail-in, with virtual support) | 9.4% |  |  |

**Views of Receiving Trial / Research Results The following questions will focus on your preferences for receiving results for both clinical trials and clinical research.**

*Which statement best applies to you regarding clinical trial / research results:*

Happy to participate, not concerned with results or info – 45.4%

I would only if getting results at some point – 29%

I would If getting results in 6m or study ending – 10.2%

I would only if getting results within 1 year of study ending – 15.4%

For clinical research studies where surveys are done or blood or other specimens are taken, if you did not get study results back within 1 year of clinical trial conclusion you would find this to be:

Acceptable as long as clinical care was met – 68.1%

Adequate time – 14.9%

Too long of a wait for feedback – 17%

The best way to learn of study results is:

Personal call – 6.9%

Letter in the mail – 41.6%

Email - 41.5%
